# *Clostridium*, a “difficile” infection that can cause a reactive arthritis

**DOI:** 10.1186/1471-2334-14-S7-P90

**Published:** 2014-10-15

**Authors:** Violeta Molagic, Anca Negru, Irina Lăpădat, Cristina Popescu, Raluca Mihăilescu, Cătălin Tilişcan, Raluca Năstase, Cristina Covaliov, Bianca Dumitrescu, Victoria Aramă

**Affiliations:** 1 National Institute for Infectious Diseases "Prof. Dr. Matei Balş", Bucharest, Romania; 2 Carol Davila University of Medicine and Pharmacy, Bucharest, Romania

## Background

*Clostridium difficile* infection (CDI) is a common cause of antibiotic-associated diarrhea and can be difficult to cure. Most patients with CDI present only colonopathy and in fewer cases extra intestinal features were described such as reactive arthritis (ReA). We present a series of cases with CDI-associated reactive arthritis (CDI-AReA), admitted between 2011-2014 in the National Institute for Infectious Diseases “Prof. Dr. Matei Balş”.

## Case report

Four patients, one male (22 years) and 3 female (median age 63 years) were diagnosed with CDI-AReA. They had monoarthritis, mild leukocytosis, elevated CRP and were treated with anti clostridial antibiotics and anti-inflammatory drugs with favorable outcome.

We include the case of a 63-year-old female admitted in May 2014 to the Adults 3 Department of INBI for watery diarrhea. One week previous to her admission, she underwent cholecystectomy and had received ceftriaxone for five days. Two days after discharge she developed 6-7 watery stools, without fever and any other symptoms. Because *Clostridium difficile* toxin EIA was positive she was admitted in our Institute with CDI and ATLAS score 2. She received vancomycin p.o.250 mg QID for 14 days. After six days she developed left knee pain and swelling. She denied any local trauma, conjunctivitis, rash, mucous membrane lesions or dysuria. The lung, cardiovascular and genito-urinary exams were normal. Musculoskeletal exam revealed left knee swelling, tenderness and painful with movement. Laboratory studies showed CRP 18.6 mg/L but normal WBC, ESR, acid uric, CPK and rheumatoid factor. Blood culture, urine culture and serology for *Chlamydia trachomatis* and *Borrelia* were negative. Stool culture was negative for *Salmonella*, *Shigella*, *Yersinia*, and *Campylobacter*. Arthrocentesis of the left knee reveled a cloudy synovial fluid with Rivalta 3+, WBC count over 50,000/cmm with 85% neutrophil, 15% mononuclear cells, fibrin 3+, high protein level, LDH 2212 IU/L and glucose 107 mg/dL. Cristal exam, gram stain and culture were negative. X-rays of the knee was negative for abnormalities and ultrasound show important joint effusion. Diagnosis of CDI-AReA was made. The patient received nonsteroidal anti-inflammatory meloxicam 15 mg/day. The diarrhea resolved quickly and no recurrent CDI occurs but the effusion persisted and slowly resolved several weeks after discharge.

## Conclusion

*Clostridium difficile* should be recognized as a rare cause of reactive arthritis. We emphasize the importance of a proper diagnosis and treatment of arthritis. It is important that clinicians avoid unnecessary antibiotic therapy for CDI-AReA.

## Consent

Written informed consent was obtained from the patients for publication of this Case report and any accompanying images. A copy of the written consent is available for review by the Editor of this journal.

